# Developing Soft Interconnected Microchannel Network for 2D Skin‐Like Actuator

**DOI:** 10.1002/advs.76995

**Published:** 2026-08-07

**Authors:** John Noee, Mohammad Akbari, Josephine Perto Justsen, Ulrich Doll, Rassoul Tabassian

**Affiliations:** ^1^ Department of Mechanical and Production Engineering Aarhus University Aarhus Denmark

**Keywords:** biomimetic, interconnected network, multi‐curvature actuation, pneumatic, soft skin

## Abstract

Soft pneumatic actuators are essential components in bioinspired and wearable systems, where flexibility, adaptability, and safe human interaction are required. However, most pneumatic designs rely on bulky chambers that limit miniaturization and prevent implementation in thin films actuation. Therefore, achieving efficient planar pneumatic actuation remains challenging. Here, a novel actuator is introduced called soft interconnected network (SIN) actuator, featuring a two‐dimensional network of interconnected microchannels embedded within an asymmetric bilayer polydimethylsiloxane (PDMS). Upon pressurization, localized strain gradients drive dual‐curvature out‐of‐plane deformation, forming a dome‐shaped motion. This skin‐like actuator exhibits large, reversible displacement and blocking force despite an overall thickness of only 2 mm. The conducted systematic geometric study shows how channel height, width, pattern resolution, and layer thickness govern actuation performance. The optimized design can achieve a large peak displacement of 30 mm under moderate pressure levels. Finally, the proposed soft actuator is implemented in a bio‐inspired prototype to mimic smooth, cyclic expansion–contraction behavior of a jellyfish bell, confirming its high potential for utilization in future bio‐inspired robots as an artificial muscle. This work establishes a generalizable platform for planar pneumatic actuation and paves the way toward next‐generation artificial skins, soft muscles, and bioinspired robots capable of 2D shell deformation.

## Graphical abstract

A soft interconnected network of microchannels is proposed to develop new pneumatic actuators for future bio‐inspired soft robots. Simultaneous stimulation of this network generates regional asymmetric strains in walls of microchannels whose interaction across the entire surface leads to bending curvatures in multidimensions. The proposed layout leads to a thin skin‐like actuator that is much thinner than conventional pneumatic actuators while providing a much larger actuation area.

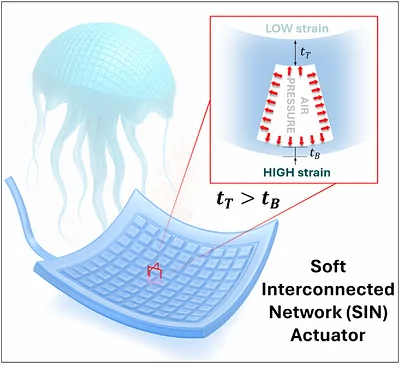

## 1 Introduction

Soft actuators have emerged as the fundamental building blocks of soft robotic systems, enabling life‐like motion, adaptability, and safe interaction with delicate environments [1, 2, 3, 4, 5, 6, 7]. Their intrinsic compliance and capability for continuous deformation make them particularly attractive for applications in biomedical devices, wearable systems, artificial muscles, and morphing structures [8, 9, 10, 11, 12]. By converting external stimuli into controllable mechanical motion, soft actuators allow robotic and bio‐inspired systems to emulate the complex behaviors observed in nature, ranging from muscular contraction and tissue expansion to adaptive surface morphologies [13, 14, 15, 16].

Among various actuation strategies, pneumatic soft actuators stand out for their reliability, simplicity, large stroke, and compatibility with biocompatible materials. They have been widely adopted in the design of grippers, crawling robots, and artificial muscles, thanks to their high power‐to‐weight ratio and inherent safety [17, 18, 19]. However, most existing pneumatic actuators rely on the volumetric expansion of bulk chambers, resulting in relatively thick and bulky geometries [20, 21, 22]. While these designs provide substantial motion in large‐scale structures, they are not well suited for thin, planar, or surface‐based applications where uniform deformation and flexibility are required in very confined space. Consequently, achieving efficient pneumatic actuation in film‐like devices remains a significant challenge.

To address this limitation, it is essential to develop pneumatic mechanisms capable of inducing large, controlled deformation within thin elastomeric sheets. Such structures are particularly appealing for artificial skin systems, wearable tactile interfaces, and bio‐mimetic actuators where compactness and softness must coexist with mechanical strength [23, 24, 25]. Despite some progress in pneumatic actuators with microchannels and soft‐lithography‐based devices, most studies have focused either on microfluidic structures exhibiting conventional one‐directional bending, or on bulk actuation, leaving the intermediate regime; thin, planar actuators capable of three‐dimensional motion are largely unexplored [26, 27, 28, 29]. Furthermore, their fabrication process involves multiple microfabrication techniques, which are very costly and cannot justify mass production.

While several planar morphing approaches, such as kirigami‐ and origami‐inspired designs, have been proposed to achieve shape reconfiguration, these methods rely on mechanical cuts or folding patterns rather than embedded pneumatic networks [30, 31]. Similarly, existing bioinspired soft robots typically achieve three‐dimensional motion by integrating multiple single‐directional pneumatic actuators arranged radially, yet such multi‐actuator assemblies remain structurally segmented and complex. These approaches collectively highlight the need for a monolithic, planar actuator capable of distributed pneumatic deformation [32, 33].

This work introduces a new concept of planar pneumatic actuation, realized through a cost‐efficient fabrication of micro‐network of interconnected air channels embedded within a thin elastomeric sheet. The actuator bridges the gap between microfluidic and bulk pneumatic systems by converting uniform pressure input into out‐of‐plane deformation via localized strain gradients. With an overall thickness of 2 mm, it produces pronounced curvature under moderate pressure through an asymmetric bilayer configuration. A key distinction of this design lies in its dual‐curvature mechanism, in which deformation occurs simultaneously along two principal directions rather than a single bending axis.

We refer to this architecture as the soft interconnected network (SIN), emphasizing its micro‐scale channel network, planar structure, and dual‐curvature deformation. The SIN actuator combines the versatility of pneumatic systems with the mechanical efficiency of thin elastic membranes, enabling skin‐like, reversible, dome‐shaped motion under uniform pressure. Owing to its low thickness, such an actuator could be applied as a skin‐like artificial muscle in future soft robotic applications. Through systematic mechanical and geometric characterization, we demonstrate how channel architecture and layer configuration govern its response, paving the way for next‐generation artificial skins, muscles, and soft morphing surfaces. Furthermore, the pneumatic actuation framework presented here establishes a robust foundation that can be extended by coupling with other stimuli mechanisms and integrated into bioinspired or underwater soft robotic systems for advanced smooth locomotion [34, 35].

## 2 Design and Fabrication

The working mechanism of the proposed actuator follows the same fundamental concept as bimorph systems, where asymmetric deformation arises from differential strain between two bonded layers. In this design, the actuator comprises a pneumatic microchannel network embedded between two PDMS layers of unequal thickness. Upon pressurization, the enclosed air channels induce local expansion in the bottom (thinner) layer, while the upper (thicker) layer constrains this motion due to its lower degree of expansion. The resulting strain mismatch generates a convex bending toward the thinner side.

Unlike conventional one‐dimensional bending actuators, the present device incorporates a two‐dimensional network of interconnected microchannels, enabling simultaneous deformation along both principal directions. This biaxial expansion produces a smooth, dome‐shaped out‐of‐plane curvature, extending the motion beyond simple beam‐like bending to a uniform shell‐like actuation. Importantly, the proposed mechanism relies on controlled strain asymmetry rather than significant bulk inflation. As a result, the overall thickness remains nearly constant during actuation. This behavior is enabled by the presence of bonding islands that constrain transverse expansion, allowing the actuator to function as a thin membrane.

As schematically illustrated in Figure 1a, the actuator converts isotropic pneumatic pressure into a controlled dome‐like deformation through localized strain gradients across its thickness. Inspired by biological locomotion, particularly the rhythmic expansion and contraction of a jellyfish bell, this actuation principle provides smooth, reversible, and spatially distributed actuation capable of mimicking soft aquatic propulsion and other bioinspired motions.

**FIGURE 1:**
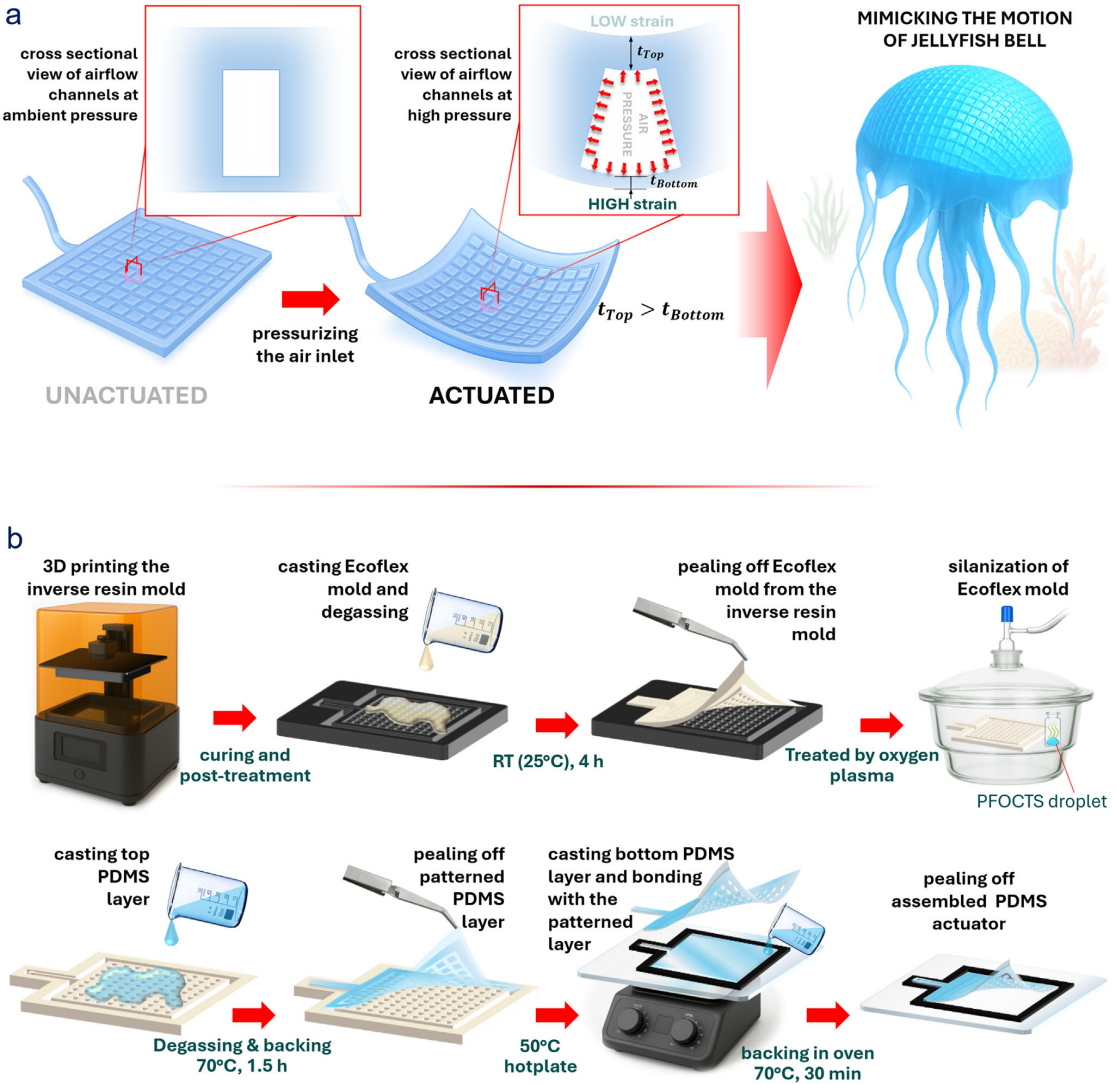
Working principle of the SIN actuator showing pressure‐induced asymmetric strain and dome‐shaped deformation. (b) Fabrication workflow including inverse‐mold 3D printing, Ecoflex casting, PDMS bonding, and final assembly.

To verify the feasibility of the proposed mechanism and guide the initial design of the actuator, a preliminary finite element simulation was conducted on the square microchannel pattern, which is described in detail in the following section. The simulation, based on a solid mechanics model implemented in COMSOL Multiphysics, provided initial estimates of the deformation behavior and the required operating pressure range. The results confirmed the expected out‐of‐plane deformation mechanism and served as a basis for selecting the geometric parameters used in fabrication.

The actuator was fabricated using PDMS (Sylgard 184, Dow Corning) owing to its optical transparency, elasticity, chemical stability, and compatibility with bioapplications and bonding processes. These properties make PDMS highly suitable for pneumatic soft actuators that undergo repeated deformation under moderate pressure while maintaining airtight integrity.

The fabricated actuator measured 7 × 7 cm with an overall thickness of approximately 2 mm. A single pneumatic inlet was incorporated, and a peripheral feeding channel, twice as wide as the internal microchannels, was designed around the network to facilitate uniform and rapid pressure distribution. Given the sub‐millimeter channel dimensions and thin PDMS layers, peel‐off and bonding remained the two most critical and challenging fabrication steps. Several approaches were evaluated, and ultimately, a two‐step molding process combined with a semi‐cured bonding technique was developed to ensure reproducibility and structural stability.

The fabrication began with a mold‐based replication process using inverse molds printed via a masked stereolithography (MSLA) 3D printer. An inverse mold was produced using UV‐curable resin with a layer thickness of 40 µm and an XY resolution of ≈ 20 µm. To prevent curing inhibition caused by interactions between residual resin components and the PDMS prepolymer, the inverse mold was thoroughly rinsed, UV‐cured for 10 min, and post‐baked at 55°C for 12 h. Subsequently, Ecoflex 00–30 (Smooth‐On) was used to produce a highly elastic intermediate mold. The two components of Ecoflex were mixed at a 1:1 weight ratio, degassed, and poured over the 3D‐printed inverse mold. After curing at room temperature for 4 h, the Ecoflex mold was peeled off and subjected to silanization to ensure smooth PDMS release. The surface was first activated using oxygen plasma (RIE, 50 W, 80 s) and then placed in a vacuum chamber for 12 h together with 100 µL of Trichloro(1H,1H,2H,2H‐perfluorooctyl)silane for vapor deposition.

Liquid PDMS was then prepared by mixing the prepolymer and curing agent at a 10:1 ratio, poured over the silanized Ecoflex mold, and degassed under vacuum. After curing at 70°C for 1.5 h, the patterned PDMS layer was carefully peeled off. A second flat PDMS layer was fabricated separately to serve as the bottom layer. For this purpose, a square frame (300 µm height) was 3D‐printed on a glass substrate to confine the liquid PDMS and ensure a uniform thickness. The glass substrate was placed on a 50°C hotplate, and once the PDMS reached a semi‐cured, tacky state, the patterned layer was gently aligned and placed on top to achieve direct bonding. The precise timing of this bonding step was critical for obtaining a seamless interface without trapped air or delamination.

The assembled actuator was post‐cured at 70°C for 30 min to enhance interfacial adhesion. A pneumatic inlet (Ø 1.5 mm) was created using a biopsy punch and connected to a flexible tube. The overall fabrication sequence is illustrated in Figure 1b, showing the two‐step molding and semi‐cured bonding process. The resulting actuator consisted of a fully enclosed microchannel network sandwiched between two PDMS membranes of unequal thickness (≈ 600 µm top and ≈ 300 µm bottom), forming a thin, optically transparent, flexible, and airtight structure suitable for repeated pneumatic testing and deformation analysis.

The fabricated samples were subsequently characterized to evaluate how the microchannel geometry and layer configuration influence their mechanical response.

## 3 Results and Discussion

### 3.1 Design and Material Characterization

To investigate how the network geometry affects the actuator's mechanical behavior, different patterns of the microchannels were designed and compared. The patterns were carefully configured to geometrically remain as symmetric as possible while ensuring smooth and uniform channel paths throughout the network. To enable a systematic comparison, three representative configurations, square, circular, and honeycomb, were selected. The selected patterns span a range of flow‐path geometries, from relatively direct paths to more tortuous networks, to capture distinct flow characteristics that influence pressure distribution. These geometries were chosen to provide a well‐balanced combination of simplicity, manufacturability, and meaningful variation in structural behavior. Figure 2a–c presents the optical image of these three patterned PDMS networks, while the magnified views in Figure 2d–f highlight the detailed topology of the interconnected air channels and bonding regions.

**FIGURE 2:**
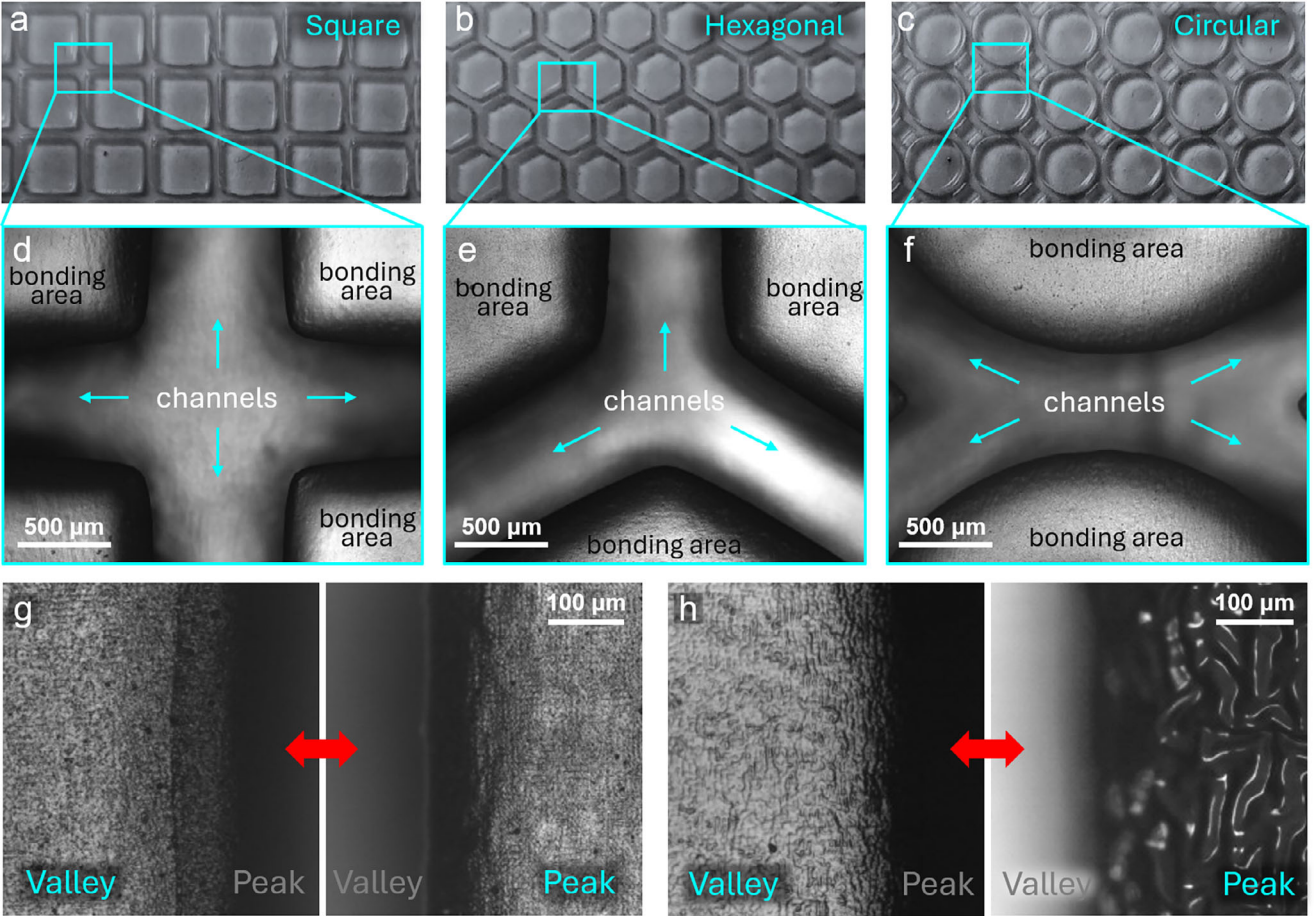
(a–c) Optical photo of the three patterned PDMS networks with: (a) square, (b) honeycomb, and (c) circular layouts. (d–f) Optical Microscope image of patterned PDMS layer with three patterns highlighting the detailed topology of the interconnected air channels and adjacent bonding regions. (g, h) Surface‐morphology comparison of (g) resin‐printed and (h) Ecoflex molds, revealing that wrinkling appears only on Ecoflex peaks (channel ceilings) while Ecoflex valleys remain smooth, preserving the bonding quality.

Each pattern exhibits a distinct regular cell geometry: the square pattern forms an orthogonal network of uniformly spaced microchannels between square cells; the honeycomb pattern consists of hexagonal cells with inclined channel walls and larger bonding areas at their junctions; and the circular layout comprises rounded cells connected through narrow necks. To preserve a constant channel width across the circular network, small rhombic intermediate cells were introduced between neighboring circles, preventing open gaps at their intersections and producing a continuous curvature and a more homogeneous network (Figure S1). This design ensured that the airflow channels remained uniform and continuous across the entire surface, similar to the other two patterns. This structural variation directly influences stress distribution and load transfer among adjacent cells, as discussed later in the mechanical tests.

The finite element simulations were subsequently refined in accordance with the final design and extended to all three geometries to provide comparative insight into their deformation responses. As shown in the displacement contour plots (Figure S2), the simulations indicate clear differences in deformation behavior among the patterns.

Since the microchannels are subjected to pressurized air and two layers of the actuator are fabricated separately, the bonding between these layers is one of the most important concerns of the suggested fabrication method. As shown in the microscopy images of patterned PDMS networks, Figure 2d–f, the suggested fabrication path led to very smooth surfaces on the bonding area with minimal morphological features that can potentially facilitate a high bonding quality with the bottom layer. Inspecting the surface morphology of the resin‐printed and Ecoflex models, however, revealed an unexpected characteristic. While both the valleys and peaks of the resin‐printed mold exhibited relatively smooth surfaces, the peaks of the Ecoflex mold, which were cast in the valleys of the resin mold, showed distinct wrinkles. However, such wrinkles were not observed in the valleys of the Ecoflex mold, corresponding to the bonding areas of the PDMS layer (Figure S3). Based on visual inspection and observations during fabrication and testing, no noticeable degradation in bonding quality between the PDMS layers was observed. The wrinkles were primarily confined to the channel ceilings and therefore mainly affected the surface roughness of the channels, as shown in Figure 2g,h.

To characterize the mechanical properties of the actuators, two mechanical tests, uniaxial tensile and bending, were conducted. For a fair and consistent comparison, all actuators were designed with identical overall dimensions and the same ratio of actuation area (channel area) to total area, while keeping the cell size, cell number, and channel width as similar as practically possible across the different patterns. To isolate the intrinsic influence of the microchannels’ geometry, the experiments were performed on the patterned layer rather than on the complete bilayer structure. A custom bending setup was employed alongside a uniaxial tensile tester to evaluate the mechanical properties along both the x and y directions (Figures S4 and S5).

The tensile tests revealed Young's modulus values ranging from 0.70 to 0.90 MPa across the three geometries, extracted from the initial linear region of the stress–strain curves. Figure 3a corresponds to the *x*‐direction and Figure 3b to the *y*‐direction. The obtained stress/strain data show very similar behavior in both *x*‐and *y*‐direction indicating nearly isotropic characteristics of patterned PDMS. This ensures comparable deformation capability in both directions and enables planar deformation with dual curvature, extending beyond conventional one‐directional bending. Among the three patterns, the square geometry appeared slightly stiffer, likely due to more direct load‐transfer paths between adjacent cells. In both the stress–strain and curvature–moment curves, all geometries behaved similarly at small deformations, but their responses gradually diverged at larger strains. The difference became more pronounced in the bending tests, which were extended to higher deformation levels where the nonlinear elastic behavior of PDMS becomes dominant (for deflection‐to‐length ratios above 0.1). Figure 3c corresponds to the bending in *x*‐direction and Figure 3d to the bending in the *y*‐direction. Three different patterns behaved almost identically at low bending strains. However, as deformation increased, nonlinear effects became dominant, and the influence of the microchannel pattern became more pronounced. In fact, the effect of patterns on the stiffness of the layer became more significant in the nonlinear deformation regime.

**FIGURE 3:**
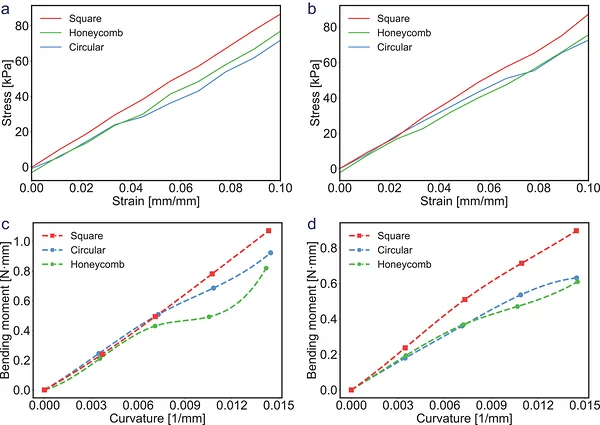
(a, b) Tensile stress–strain response of the three patterned PDMS networks (square, honeycomb, and circular) in (a) *x*‐direction and (b) *y*‐direction. (c, d) Bending moment–curvature curves calculated at the fixed end in (c) x direction and (d) y direction, showing the highest bending resistance for the square pattern, followed by the circular and honeycomb designs.

### 3.2 Actuation Performance

#### 3.2.1 Pattern Comparison

Following the mechanical characterization, the actuation performance of the samples was investigated through a series of pneumatic tests, focusing on the transient, cyclic, and dynamic response as well as the blocking‐force characteristics.

Figure 4a–c illustrates the deformation of actuators with different network geometries under identical pneumatic loading. The images compare the unpressurized (OFF) and pressurized (ON) states, clearly showing the distinct upward bending that occurs upon activation and confirming the bidirectional curvature mechanism described earlier.

**FIGURE 4:**
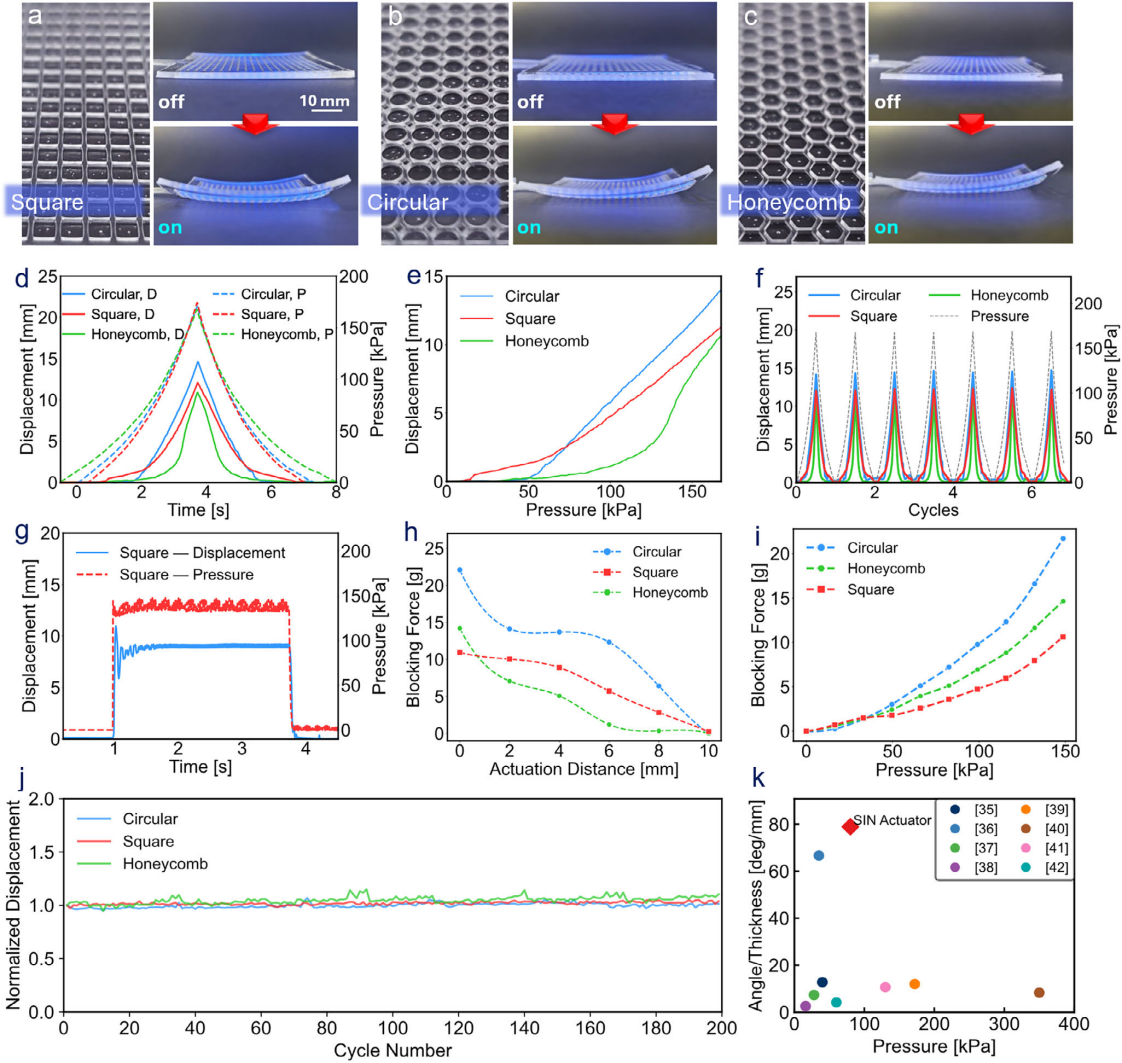
(a–c) Optical images and side‐view actuation snapshots of SIN actuators with (a) square, (b) circular, and (c) honeycomb channel networks, showing their off and on states. (d) Transient displacement response and corresponding pressure profile for the three geometries under a single pressurization–depressurization cycle. (e) pressure–displacement curves highlighting the higher actuation amplitude of the square pattern. (f) displacement and pressure signals over multiple actuation cycles demonstrating the repeatability of each design. (g) transient response of the square design under a square‐pulse pressure input. (h) Blocking force as a function of actuation distance, showing reduced force with increasing probe–surface gap. (i) Blocking force as a function of applied pressure for all geometries. (j) Durability performance of the actuators under cyclic pneumatic loading. Normalized peak displacement as a function of cycle number for circular, square, and honeycomb geometries. (k) Performance comparison of the SIN actuator with representative pneumatic soft actuators from the literature. The thickness‐normalized bending angle (bending angle per unit thickness) is used as a size‐independent metric of bending capability.

To evaluate the actuation performance under pneumatic loading, a custom experimental setup was developed and operated via LabVIEW. The working pressure was applied by a syringe pump rising the pressure from 0 to the desired target value. A pressure sensor was used in the air hose to provide feedback for the syringe pump as well as real‐time pressure information. The resulting deformation was recorded by a laser displacement sensor. The schematic diagram of the measurement setup is presented in Figure S6.

Two boundary configurations were examined: free and clamped. In the free configuration, the actuator was placed on a flat surface, and the displacement before and after pressurization was measured at one of the corners. In the clamped configuration, the actuator was fixed along one edge, and the displacement was measured at the midpoint of the opposite edge. This test was performed along both the *x*‐and *y*‐directions to investigate pure displacement in either of the directions. This setup enabled direct quantification of curvature in each principal direction, allowing a comparative evaluation of the bidirectional bending behavior. The experimental setups for both configurations are shown in Figure S7.

The corresponding pressure–time and displacement–time curves for the free end measurement are shown in Figure 4d. Although identical settings were used for all tests regarding the maximum target pressure and the speed of the syringe pump motor, the actual pressure profiles were not perfectly identical. Pneumatic systems inherently involve minor control fluctuations, leading to small tolerances in peak pressure values. In addition, variations in the geometry and arrangement of the microchannel networks can alter the airflow paths and slightly affect the local pressure distribution within each actuator. Nevertheless, all efforts were made to maintain consistent input pressure to ensure a fair comparison among the samples.

Under the same maximum pressure (∼170 kPa), the circular pattern produced the largest peak displacement (≈ 14.6 mm), followed by the square (≈ 12.1 mm) and honeycomb (≈ 10.9 mm) geometries. The honeycomb structure, which has the largest unit cell area based on the defined geometric parameters, exhibited the slowest response. This behavior may be attributed to its geometric characteristics, including a more tortuous channel layout and frequent changes in flow direction, which are expected to increase resistance to transient airflow. In contrast, the circular pattern with its finely distributed microchannels showed a rapid actuation after an initial lag. This transient delay may be caused by the rhombic intermediate cells, which locally restrict airflow and delay pressure equalization across the network. The square design, featuring straight and uniform channels, displayed an intermediate behavior with a smooth and balanced transition. These geometric distinctions collectively explain the actuation profiles observed in Figure 4d.

The corresponding displacement–time and pressure–time plots for the x and y orientations in the clamped configuration are provided in the Figure S8. The response along the y direction followed a similar trend to that observed in the free configuration, whereas the displacements in the *x* direction were much closer among the different geometries. This difference may be related to the test configuration: in the *y*‐direction tests, the air inlet was positioned behind the clamped edge, while in the *x*‐direction tests, the inlet was located along the lateral edge of the actuator, introducing additional unintended constraints. Although efforts were made to minimize this effect during testing, its influence on the measured displacements cannot be completely ruled out. At the same time, the tests clearly revealed two distinct curvatures developing along the orthogonal directions, confirming the bidirectional bending behavior of the actuators.

Plotting the displacement as a function of applied pressure (Figure 4e) effectively decouples the actuator's intrinsic mechanical response from the transient characteristics of the input pressure, allowing a more direct comparison between geometries. The slope of the displacement–pressure curve represents the actuator's sensitivity to the applied stimulus. Two distinct phases can be identified: an initial filling phase, dominated by air‐channel inflation, followed by a deformation phase characterized by in‐plane expansion and out‐of‐plane bending. The circular pattern displays the steepest and most linear slope, indicating the highest sensitivity to pressure; the square pattern follows a moderate linear trend, beginning with a mild slope and then increasing progressively, while the honeycomb structure exhibits a delayed and nonlinear response.

To further characterize the time‐dependent response of the actuators, hysteresis behavior was evaluated through displacement–pressure loading–unloading cycles. The corresponding results are provided in the Figure S9. The enclosed area of the hysteresis loops provides a qualitative measure of energy dissipation, while the separation between the loading and unloading paths reflects time‐dependent effects such as viscoelasticity and flow‐induced resistance. The honeycomb actuator shows larger path separation but operates over a smaller pressure range than the square and circular designs. As a result, the enclosed hysteresis area, and therefore the level of energy dissipation, remains comparable. However, the increased separation indicates greater lag and higher resistance to fluid flow within the honeycomb channel network. These observations are consistent with earlier trends and further support the role of internal geometry in governing actuation behavior.

We evaluated the repeatability and stability of the actuators by performing cyclic pneumatic loading tests. As shown in Figure 4f, the displacement response was consistently reproduced across multiple loading–unloading cycles, following a nearly identical trend in each cycle, and without any degradation in actuator performance. These results indicate fully reversible actuation, so that after each stimulation the actuator can fully retrieve its initial shape and get ready for the next actuation cycle.

The actuator's dynamic behavior was also analyzed through its transient displacement response to a square‐wave pneumatic pressure input. A dedicated pneumatic setup, consisting of a miniature air pump, solenoid valves, and an electronic control unit (Figure S10), was used to generate a near‐ideal square‐wave pressure through rapid pressurization and exhaust. The applied pressure signals were consistent across all actuator designs, ensuring that the effects of pressure and geometry can be decoupled. Figure 4g presents the representative pulse‐response behavior for the square pattern, while the corresponding results for the circular and honeycomb geometries are provided in the Figure S11. Both the square and circular actuators exhibited a noticeable overshoot followed by short‐lived oscillations after the initial pressurization, which can be attributed to the rapid dynamic response of the structure. In contrast, the honeycomb actuator responded significantly more slowly, requiring a relatively long time to reach the steady state. This behavior suggests a higher resistance to transient airflow in the honeycomb network, which may originate from the more tortuous flow paths and frequent changes in flow direction within the channel network. Therefore, it is not well suited for applications that require very fast actuation. In comparison, the square pattern provides relatively straight and shorter flow paths and exhibits faster dynamic behavior, making it suitable for high‐speed actuation applications.

In addition to displacement analysis, the blocking force developed during pneumatic actuation was evaluated. The measurements were conducted using a syringe pump at a working pressure of 150 kPa. A load cell was employed to record the force applied at the central point of the actuator. During the tests, the actuators were placed on a flat surface (free configuration), and the probe tip of the load cell was positioned at predefined distances from the actuator's initial surface. For each test, the force generated upon deformation and contact with the probe was recorded.

Figure 4h presents the measured blocking force for the three patterns as a function of the probe's initial distance from the actuator surface. At zero gap, where the probe is in full contact with the actuator surface, most of the supplied pneumatic energy is directly transmitted to the probe, producing a substantial blocking force. The localized constraint imposed by the probe also alters the deformation mode of the membrane, redistributing part of the strain energy to the surrounding regions. As the initial gap between the probe and the actuator surface increases, a larger portion of the pneumatic energy is consumed to overcome the stiffness of the actuator and deform it before contact, leaving less available to be transmitted as blocking force upon contact. This deformation energy consumption continues until the actuator reaches its ultimate deformation and no energy is left to be transmitted to external objects as blocking force. Consequently, the measured blocking force decreases steadily with increasing gap and approaches zero near the 10 mm distance, where the supplied energy is almost entirely stored or dissipated through elastic deformation and internal losses within the actuator. This evolution suggests that actuators capable of larger deformation (such as the circular pattern) convert a greater portion of the input pneumatic energy into external reaction, consistent with their higher displacement and blocking force.

To further quantify this behavior, Figure 4i shows the blocking force as a function of the applied pressure under the zero‐gap (direct contact) condition where the pressure gradually increased from 0 to the maximum value. The same measurement is conducted for a 2 mm gap, with results provided in the Figure S12. The slope of each curve can reflect the actuator's pneumatic efficiency; a higher slope implies a greater ability of the actuator to convert input pressure into mechanical output force under the given conditions. In both cases, the circular pattern exhibits the highest slope, indicating greater conversion efficiency and load‐bearing capability. The relative performance of the square and honeycomb geometries depends on the contact regime: under direct contact, the honeycomb actuator shows a slightly higher force due to its stiffer local support, whereas at a 2 mm initial gap, the square pattern becomes more efficient, benefiting from its straighter airflow paths and reduced pressure losses.

In addition, durability tests were conducted to evaluate the reliability of the actuators under a clamped configuration, ensuring stable boundary conditions during cyclic loading. As shown in Figure 4j, the normalized peak displacement remains stable over 200 pressurization–depressurization cycles for all geometries, with no evidence of degradation in actuation amplitude. The mean normalized peak displacement remains close to unity, with small standard deviations (≈1%–3%), indicating consistent and repeatable performance. These results demonstrate the robustness of the actuators under repeated operation.

To place the performance of the proposed actuator in a broader context, a comparative analysis with representative pneumatic soft actuators reported in the literature [36, 37, 38, 39, 40, 41, 42, 43] is presented in Figure 4k. Direct comparison is challenging due to fundamental differences in geometry and operating mechanisms. Increasing the channel/chamber height increases the bending moment through a larger pressure‐loaded area and a longer lever arm, thereby enhancing displacement and blocking force, but at the cost of increased actuator thickness (see Figure S13). To account for these differences, the thickness‐normalized bending angle, defined as the bending angle (measured in the *xz* plane for the SIN actuator) divided by the actuator thickness, is used as a size‐independent metric for fair comparison across designs. As shown in Figure 4k, the SIN actuator achieves a high normalized bending angle (79 deg/mm) at moderate pressure levels (∼80 kPa), indicating efficient deformation within a thin structure. In contrast, many conventional actuators require either higher pressures or greater structural thicknesses to reach comparable bending angles. A summary of the literature data used for this comparison is provided in Table S1. These results support the effectiveness of the microchannel‐based architecture in achieving large deformation while maintaining a compact form factor.

Overall, the results highlight that the actuation behavior strongly depends on the internal channel geometry and its ability to distribute pressure uniformly across the structure. Among the three examined layouts, the circular pattern demonstrated the largest deformation, the square design provided the fastest and most efficient response, and the honeycomb pattern exhibited the slowest and stiffest behavior. These differences originate from the interplay between airflow path length, channel connectivity, and structural stiffness. The analysis also indicates that the actuation efficiency and blocking force can be tuned by adjusting geometrical features such as channel width and height, which are examined in detail in the following section.

#### 3.2.2 Parametric Study

To gain deeper insight into how geometric design parameters influence the actuation behavior, a systematic study was conducted. The analysis examined how variations in four key geometrical factors, channel height, channel width, PDMS layer thickness, and pattern resolution affect the actuator's displacement and blocking force. In each case, all other design variables were kept constant to isolate the effect of the selected parameter. All parametric variations were performed using the square pattern as the base geometry, owing to its fast, smooth, and efficient mechanical response.

Before presenting the quantitative results, Figure 5a,b visually illustrates the deformation sequence of a representative actuator with the best geometric features during pressurization and release. Under increasing pressure (Figure 5a), the actuator gradually bends upward, reaching its maximum curvature at the peak pressure. Upon pressure release (Figure 5b), the structure smoothly returns to its initial flat state, demonstrating reversible deformation and good structural integrity. These snapshots qualitatively confirm the actuator's repeatable bending behavior. A real‐time video of the actuation is also available in Video S1.

**FIGURE 5:**
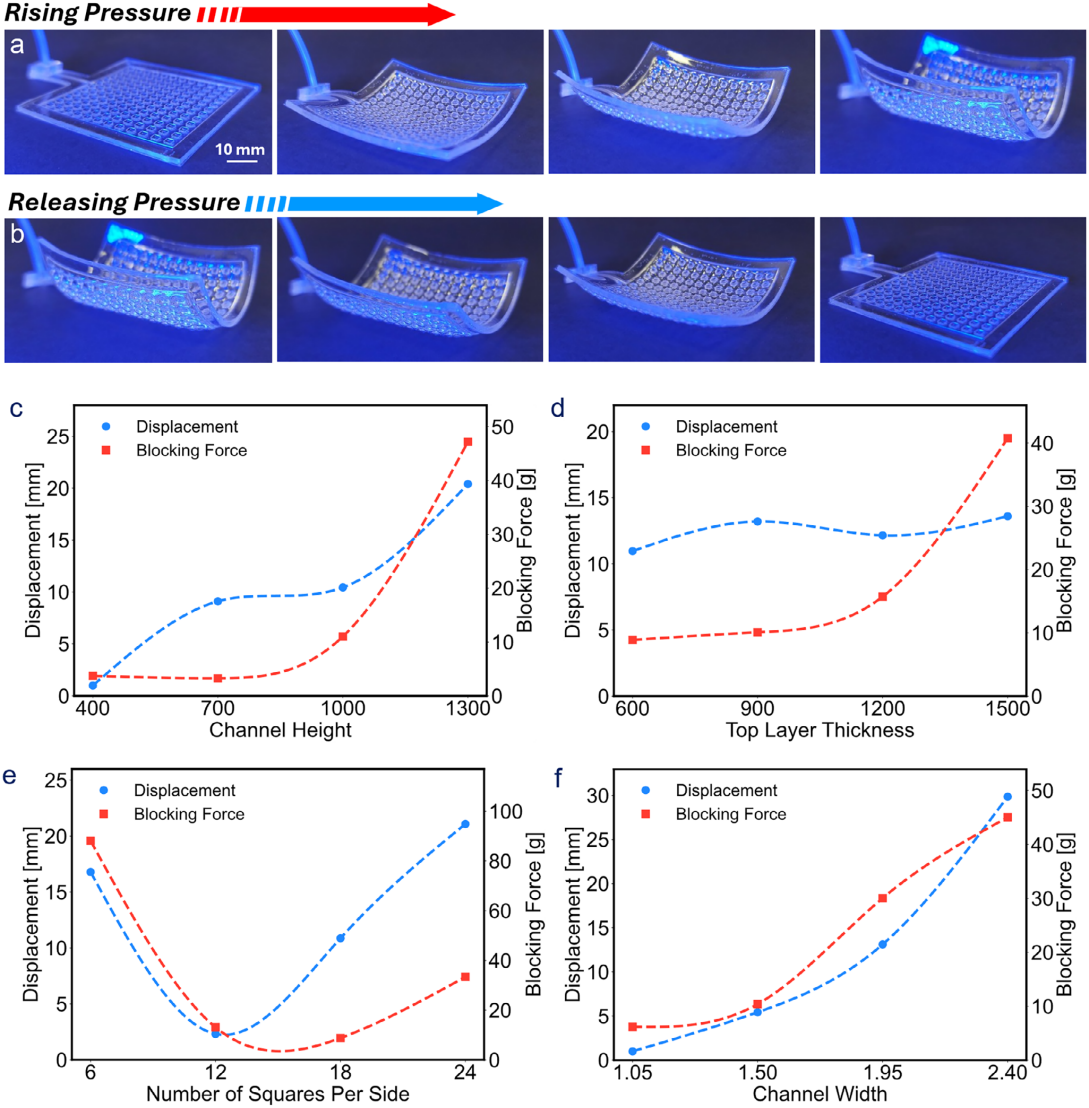
(a, b) Sequential snapshots of a square‐patterned SIN actuator during (a) pressurization and (b) depressurization, demonstrating its reversible dual‐curvature deformation. (c–f) Influence of key geometric parameters on actuation performance: (c) channel height, (d) top‐layer thickness, (e) pattern resolution (number of squares per side), and (f) channel width.

To begin, the same square‐pattern actuator used in the performance analysis was selected as the reference design. This actuator consists of 18 × 18 array of square cells, with a channel height of 1000 µm, a channel width of 700 µm, and PDMS layer thicknesses of 600 µm (top) and 300 µm (bottom). To examine the effect of channel height, three additional actuators were fabricated with channel heights of 400, 700, and 1300 µm. All actuators were tested under identical working pressures of 159 kPa for the displacement measurements and 152 kPa for the blocking‐force tests. The corresponding results are presented in Figure 5c. As expected, the channel height shows a direct correlation with both the displacement and the blocking force: increasing the channel height leads to a proportional increase in both quantities. At the upper end of the curve, the slope of both displacement and blocking‐force plots increases, indicating a transition to a more nonlinear response regime. This behavior can be attributed to a change in the deformation mechanism, where larger input energy leads to greater strain and geometric nonlinearities, amplifying the overall actuation response.

The next parameter examined was the thickness ratio of the PDMS layers. The actuator was expected to be more sensitive to the thickness of the lower (thin) layer, which functions as the active component, whereas the thicker layer acts as the passive one. However, due to fabrication limitations, variation of thickness distribution was implemented by changing the thickness of the top (thick) layer. To evaluate its influence, the bottom layer was kept constant while the top layer thickness was increased to 900, 1200, and 1500 µm. The working pressures for the displacement and blocking‐force tests were 164 and 160 kPa, respectively. The plotted results in Figure 5d show that the blocking force increased steadily with the top‐layer thickness as expected. However, the displacement showed a very gradual increasing trend, with a reduced rate of growth as the top‐layer thickness increased. This is because two contradictory effects occur when the thickness of the top (thick) PDMS layer increases. Increasing the passive‐layer thickness enhances the top–bottom asymmetry and strengthens the deformation mechanism but simultaneously increases the global stiffness and constrains bending. As the top layer becomes thicker, this constraint becomes more pronounced. The interplay between these opposing effects results in the mild overall increase observed in the displacement curve. Reducing the thickness of the bottom layer, rather than thickening the top one, would likely yield a more pronounced actuation response, as the thickness of the bottom (thin) layer has much less contribution to the overall stiffness of the actuator. However, such modification introduces significant fabrication challenges.

The next geometric parameter examined was the effect of pattern resolution (fineness) on actuator performance. To isolate this factor, the ratio of channel width to bonding length was kept constant while both parameters were scaled up or down simultaneously. Consequently, the number of cells along the horizontal and vertical directions changed proportionally. Using this approach, new molds were designed to produce actuators with different cell densities (fine to coarse networks), while keeping the total bonding area constant at 2025 mm^2^, and hence the same total actuation area. Three additional actuators were fabricated with 6 × 6, 12 × 12, and 24 × 24 cell configurations (Figure S14). As shown in Figure 5e, both the displacement and blocking‐force curves exhibit a mid‐range minimum in their overall trend. This nonmonotonic trend also originates from the competition between two opposing mechanisms: structural compliance and pneumatic transmission efficiency. As the pattern becomes finer and the size of individual cells shrinks, the pressure distribution across the actuator surface becomes more uniform, and the structure gains flexibility, facilitating larger deformations. In contrast, when the pattern becomes coarser and channels widen, the flow resistance decreases, and the pressure propagates more rapidly, leading to a more efficient transfer of pneumatic energy to each cell. The overall actuator response is governed by the interplay of these two effects. At intermediate pattern densities, the opposing mechanisms nearly balance each other, resulting in reduced actuation performance. At both ends of the range, however, one mechanism dominates and enhances the response; the finer network favors larger displacement due to its higher flexibility, whereas the coarser network yields higher blocking force thanks to its greater stiffness and load‐bearing capacity. Consistent with this interpretation, the actuator with the coarsest pattern exhibited the highest blocking force among all fabricated samples, reaching approximately 88 g.

To investigate the effect of channel width on actuator performance, three additional actuators were fabricated, each consisting of 12 × 12 square cells, in addition to the one used in the pattern‐resolution study (Figure S15). The smaller number of cells (12 × 12 compared with 18 × 18) allowed the use of a wider range of channel widths, which were then systematically examined and compared. In all samples, the sum of the channel width and the bonding length was kept constant to isolate the influence of channel width. The actuators were tested under working pressures of 78 kPa for the displacement measurement and 81 kPa for the blocking‐force test. These pressures were approximately half of those used in the previous experiments, as this group of actuators exhibited much higher sensitivity to the input pressure. Even at such lower pressures, remarkably large deformations were observed. As shown in Figure 5f, both the displacement and blocking force increase with the increase of channel width. This rise is particularly pronounced in the displacement response: The actuator with a channel width of 2.4 mm showed the largest deformation (≈ 30 mm) among all tested samples, despite being driven at a much lower input pressure. Such a level of actuation can generate an estimated elastic power density of 4 W/kg and an elastic energy efficiency of 72% based on the validated finite element simulation (see Supporting Information). This trend is expected, as widening the channels increases the effective actuation area while decreasing the flow resistance, thereby enhancing the pneumatic energy delivered per unit area. It should also be noted that decreasing the bonding area between top and bottom layers can compromise the structural stability and introduce fabrication challenges. Therefore, the channel width cannot be increased indefinitely, as excessive widening can weaken the bonding regions and reduce the actuator's mechanical reliability.

Overall, the geometric analysis reveals that the actuation performance can be effectively tuned by adjusting structural dimensions across multiple scales. Increasing the channel height or width enhances both deformation and blocking force by enlarging the effective pneumatic volume and actuation area. The PDMS layer thickness primarily controls bending stiffness through top–bottom asymmetry, while the pattern resolution determines the balance between structural flexibility and pneumatic transmission efficiency. Collectively, these findings highlight that geometric optimization provides a powerful means of tailoring actuator performance for specific requirements such as high displacement, high force, or fast response.

### 3.3 Jellyfish‐Inspired Motion

Jellyfish‐inspired soft robots have been of great interest due to their efficient propulsion mechanisms in fluid environments [44, 45]. To illustrate the applicability of the developed actuator, we constructed a bioinspired prototype resembling a jellyfish bell. The structure consisted of a central circular SIN actuator surrounded by a peripheral sinusoidal fin. The central actuator employed a square‐patterned channel network with geometric dimensions derived from the optimized design parameters identified in the previous section.

In natural jellyfish propulsion, locomotion is generated through an asymmetric cyclic contraction–expansion of the bell muscle, characterized by a rapid contraction followed by a slower relaxation. To qualitatively reproduce this asymmetric motion, an asymmetric pneumatic actuation profile was applied using the same setup described in Figure S10. Under asymmetric cyclic pressurization, the actuator sequentially curved and flattened, producing a pulsed dome‐like deformation that induces surrounding fluid motion and resembles the rhythmic cycle of a jellyfish bell. The corresponding deformation cycle of the SIN prototype under cyclic pneumatic input is illustrated in Figure 6a–f. A real‐time video of the motion is provided in Video S2.

**FIGURE 6:**
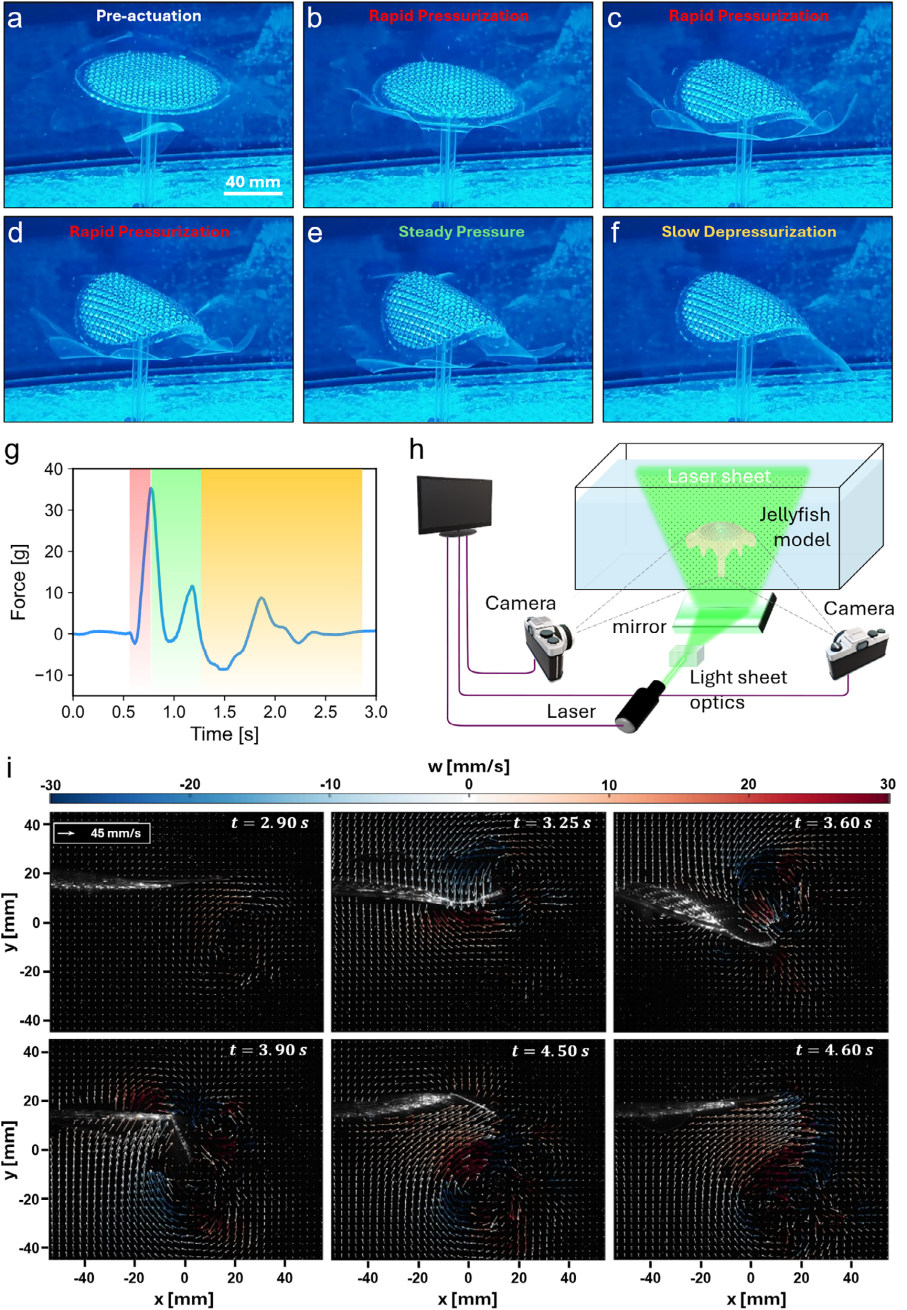
(a–f) Sequential underwater actuation of the SIN prototype demonstrating biomimetic smooth deformation resembling jellyfish bell motion, (g) measured propulsion force within a full cycle of underwater actuation, where the white region corresponds to the pre‐actuation state, the pink region to the rapid pressurization phase, the green region to the steady pressure phase, and the yellow region to the slow depressurization phase, (h) schematic of the particle image velocimetry (PIV) measurement setup. (i) sequential velocity vector fields obtained from the PIV measurements during one underwater actuation cycle.

This demonstration does not aim to realize a fully functional jellyfish‐like robot but rather highlights the SIN actuator's potential to achieve biomimetic motions through spatially distributed pneumatic stimulation. The observed motion was smooth, reversible, and repeatable over multiple cycles, confirming the structural integrity of the thin PDMS membranes. To further investigate the propulsion functionality of the jellyfish actuator, additional quantitative and flow characterization experiments were conducted.

The propulsion force generated by the actuator was measured using a modified setup in which the underwater holder was mechanically connected to a load cell out of water (see schematic in Figure S16). The measured force was converted to the corresponding axial thrust based on the lever geometry as follows:

$$T=\frac{L_{1}}{L_{2}}\;F_{LC}$$

where ***T*** is the propulsion force, ***F_LC_*** is the measured force on the load cell, and ***L_1_*** and ***L_2_*** are the lengths of the holder arms. As shown in Figure 6g, the actuator generates a peak positive thrust of approximately 35 g during rapid pressurization, followed by a smaller negative force during relaxation. The pink region corresponds to a rapid downward actuation caused by instantaneous pressurization, resulting in the maximum forward thrust. In the green region, pressure continues to be applied; however, the downward actuation slows as the actuator approaches its maximum displacement, leading to a reduction in the generated positive thrust. The yellow region represents a slow depressurization stage, during which the actuator returns to its original shape through upward motion, thereby producing a negative thrust. Notably, since the net thrust is positive, the actuator is capable of generating net forward propulsion over a full actuation cycle. To benchmark the performance of the proposed jellyfish‐inspired SIN actuator, we further compared its thrust characteristics with those of previously reported jellyfish‐inspired robotic systems [45, 46, 47, 48]. The comparison indicates that the proposed actuator achieves highly competitive thrust performance, particularly when normalized by the bell diameter. A summary of the literature data used for this comparison is provided in Table S2.

To better understand and analyze the actuator–fluid interaction, stereoscopic particle image velocimetry (S‐PIV) was also conducted, and three‐component flow fields were measured. The flow was seeded with tracer particles and illuminated by a pulsed laser, while two cameras in a stereo configuration captured the scattered light. The acquired images were processed using a multi‐pass correlation algorithm to obtain velocity vector fields, followed by standard filtering and denoising procedures to improve data quality. The experimental setup is shown in Figure 6h.

Video S3 shows the flow fields around the jellyfish prototype during multiple contraction–relaxation cycles. Selective frames of one full cycle are presented in Figure 6i. The arrows represent the in‐plane velocity components (*u*, *v*), while the color field indicates the out‐of‐plane component *w*. Initially, the flow field is nearly at rest, although weak residual motion from the preceding cycle remains visible. As the prototype contracts, the surrounding fluid is accelerated, and vortical structures develop near the peripheral fin before being convected downstream. The alternating positive and negative values of *w* indicate the three‐dimensional nature of the induced flow. These results demonstrate that the actuator actively drives fluid motion, generates momentum exchange with the surrounding water, and is capable of producing measurable thrust.

The presented jellyfish‐inspired prototype further highlights the SIN architecture's ability to generate distributed, skin‐like deformations within a compact configuration. Such capabilities open new avenues toward soft robotic systems, adaptive morphing surfaces, and bioinspired locomotion and aquatic propulsion.

## 4 Conclusion

In this study, we introduced the SIN actuator (a micro dual‐curvature planar network actuator) that establishes a new paradigm of thin pneumatic soft actuation. By embedding a micro‐network of interconnected air channels between two asymmetric PDMS layers, the actuator converts uniform pressure into smooth, dome‐like deformation through localized strain gradients. Systematic performance and geometric studies revealed how both the microchannel pattern (square, honeycomb, circular) and structural parameters, including channel height, channel width, layer thickness, and pattern resolution, govern the actuator's displacement and blocking force. The results demonstrated that the SIN actuator can achieve large, reversible, and biaxial curvature within a compact 2‐mm‐thick elastomeric sheet while maintaining mechanical stability and airtight integrity. Beyond its fundamental performance, the jellyfish‐inspired demonstration validated the actuator's ability to generate spatially distributed, biologically relevant motion using a single pneumatic input. This capability highlights its potential for integration into artificial skins, soft muscle networks, morphing surfaces, and aquatic robotic systems that require smooth and distributed actuation.

Looking forward, the presented pneumatic platform provides a robust foundation for future extensions toward network actuation schemes, enabling higher controllability and more compact integration beyond the current limitations of single actuators. Such developments could lead to next‐generation soft robotic systems that combine multiple actuation modes and bridge the gap between natural biological motion and engineered adaptability.

## 5 Experimental Section

### 5.1 Materials

Polydimethylsiloxane (PDMS, Sylgard 184, Dow Corning) was used as the primary elastomer owing to its optical transparency, elasticity, and biocompatibility. Ecoflex 00–30 (Smooth‐On) was employed as the intermediate mold material due to its high flexibility and easy demolding characteristics. Trichloro(1H,1H,2H,2H‐perfluorooctyl)silane (Sigma–Aldrich) served as the silanization agent. Isopropanol (IPA) and deionized water were used for cleaning and rinsing. All materials were used as received without further purification.

### 5.2 Mold Fabrication Via MSLA 3D Printing

Inverse molds defining the microchannel geometry were fabricated using a high‐resolution masked stereolithography (MSLA) 3D printer (Elegoo Saturn 3 Ultra) equipped with a 12K LCD display, providing an XY resolution of 19 × 24 µm and a layer thickness of 40 µm. A UV‐curable resin (Elegoo 8K Standard) was used as the printing material. After printing, the molds were rinsed with isopropanol for 7 min to remove uncured resin, dried at room temperature, UV‐cured for 10 min, and post‐baked at 55°C for 12 h to eliminate residual monomers that could inhibit PDMS curing.

### 5.3 Preparation of the Ecoflex Intermediate Mold

A soft intermediate mold was prepared by casting Ecoflex 00–30 mixed at a 1:1 weight ratio (parts A:B). The mixture was degassed under vacuum for 5 min and poured over the 3D‐printed resin mold. After curing for 4 h at room temperature, the Ecoflex mold was demolded and prepared for surface treatment. To ensure smooth PDMS release, the Ecoflex surface was first activated using oxygen plasma (RIE, 50 W, 80 s) and then placed in a vacuum chamber with 100 µL of Trichloro(1H,1H,2H,2H‐perfluorooctyl)silane for 12 h to achieve complete vapor‐phase silanization.

### 5.4 PDMS Casting and Curing

PDMS prepolymer and curing agent were mixed at a 10:1 weight ratio, stirred thoroughly, and degassed under vacuum for 10 min. The mixture was poured onto the silanized Ecoflex mold, degassed again, and cured at 70°C for 1.5 h to form the patterned layer. After curing, the PDMS was carefully peeled off to obtain the microchannel‐embedded top layer. A second flat PDMS layer was fabricated separately by pouring the same PDMS mixture into a 3D‐printed frame (height ≈ 300 µm) placed on a glass substrate to control the layer thickness. This layer served as the bottom membrane.

### 5.5 Bonding Process

The patterned and flat PDMS layers were bonded using a semi‐cured (tacky) PDMS technique. The flat layer was pre‐heated on a 50°C hotplate until it reached a semi‐cured state, after which the patterned layer was gently aligned and placed on top. The assembled device was post‐cured at 70°C for 30 min to strengthen interfacial adhesion. Precise timing of the bonding step was crucial to avoid over‐curing (resulting in poor adhesion) or under‐curing (causing air entrapment and partial filling of microchannels). Successful bonding produced a seamless, air‐tight interface without any delamination.

### 5.6 Pneumatic Inlet and Tubing

A pneumatic inlet (Ø 1.5 mm) was created using a biopsy punch in the patterned layer. After bonding, an additional 5 mm‐thick PDMS pad was added around the inlet and re‐punched using a Ø 4 mm punch to provide mechanical support. The pneumatic line was connected using a flexible polyurethane microtube (outer diameter 4 mm, inner diameter 1.5 mm). The tube fit tightly into the punched port, ensuring a leak‐free seal without adhesive.

### 5.7 PIV Measurement

To conduct the measurement, the jellyfish actuator was immersed in a water‐filled tank (100 cm × 40 cm × 40 cm). The model and associated hosing were secured via a holder connected to the tank's rear wall. Three‐component flow field measurements were performed using stereoscopic particle image velocimetry (S‐PIV). A planar area coinciding with the model's symmetry axis was illuminated from below by a Litron NL 120‐20 double‐cavity laser. The flow was seeded with 60 µm spherical polyamide particles; scattered light was captured by two LaVision Imager CX3‐5 cameras. Each camera was equipped with f = 50/1.8 lenses and 532 nm bandpass filters (10 nm bandwidth), mounted on Scheimpflug adapters to maintain focus across the tilted focal plane. Cameras were positioned in an angular stereo configuration with a total inter‐camera angle of 47.06°. Calibration was performed using a pinhole fit model, resulting in a dewarped measurement plane of 115.5 mm × 97.4 mm and a scale factor of 21.11 pixels/mm. The system operated in double‐frame mode with a pulse separation of 6 ms at a repetition rate of 20 Hz. Data acquisition yielded 500 double‐frames over 25 s, capturing seven full actuation cycles. Vector fields were calculated using LaVision DaVis software via a multi‐pass correlation scheme, concluding with a final interrogation window of 24 × 24 pixels with 50% overlap, resulting in a velocity grid spacing of 1.14 mm. Post‐processing of the vector fields included a median outlier filter to remove spurious vectors and anisotropic denoising to enhance the signal‐to‐noise ratio.

Further technical details and schematic illustrations of the experimental setup and imaging configuration can be found in the Supporting Information. In this manuscript, AI tools were used merely for English editing.

## Author Contributions

**John Noee**: conceptualization, investigation, methodology, validation, software, writing – original draft, visualization, formal analysis, data curation. **Mohammad Akbari**: methodology, writing – review and editing, conceptualization. **Josephine Perto Justsen**: methodology. **Ulrich Doll**: methodology, writing – review and editing. **Rassoul Tabassian**: conceptualization, methodology, writing – review and editing, resources, supervision, project administration, visualization, funding acquisition.

## Funding

This work was supported by the Novo Nordisk Foundation, NNF22OC0072014.

## Conflicts of Interest

The authors declare no conflict of interest. The funding agency had no role in the study design, data collection and analysis, decision to publish, or preparation of the manuscript.

## Supporting information




**Supporting File 1**: advs76995‐sup‐0001‐SuppMat.docx.

**Supporting File 2**: advs76995‐sup‐0002‐VideoS1‐S3.zip.

## Data Availability

The data that support the findings of this study are available from the corresponding author upon reasonable request.
