# Supplementary material for: Developing Soft Interconnected Microchannel Network for 2D Skin‐Like Actuator
Source: Adv Sci (Weinh). 2026 Aug 7;13(60):e76995. doi: 10.1002/advs.76995 (PMC13449759; doi:10.1002/advs.76995)
Supplement: Supplementary file 1 [file ADVS-13-e76995-s002.docx]

Supporting Information

Developing Soft Interconnected Microchannel Network for 2D Skin-like Actuator

John Noee, Mohammad Akbari, Josephine Perto Justsen, Ulrich Doll, and Rassoul Tabassian*

**S.1 Experimental Setup and Characterization**

Pneumatic actuation tests were performed using a custom pressure-control system comprising a syringe pump (bipolar stepper motor, a lead screw, linear guide rods, and syringe) and a differential pressure sensor (MPX5500DP, NXP), capable of delivering pressures up to 250 kPa.
For cyclic square-wave pressure actuation, a portable electric air compressor (150 psi max), solenoid valves (2V025-08, Airtac), and a microcontroller (Arduino UNO) were employed. Pressure data were acquired using the same type of differential pressure sensors.
Out-of-plane displacement and dome height were measured using a laser displacement sensor (optoNCDT 1220, Micro-Epsilon) positioned above the actuator, with data recorded in LabVIEW 2023. Blocking-force measurements were performed by contacting the actuator with a load cell (FUTEK LSB201) and recording the reaction force during pressurization. All experiments were carried out at room temperature (≈ 22 °C).

Tensile tests were performed using a universal testing machine (Zwick/Roell Z050, 50 kN capacity) equipped with a load cell (AST KAP-TC, 60 kN, 2.0 mV/V, Class 0.05). Bending tests employed the same stepper-motor setup, load cell, and laser displacement sensor.

**S.2 Imaging and Visualization**

Microscopic analyses were performed using an upright optical microscope (Zeiss Axio Scope.A1, Carl Zeiss Microscopy GmbH, Germany) equipped with a Deltapix Infinity camera. The microscope provided magnifications from 5× to 100×, supporting bright-field imaging for surface morphology characterization.

**Figure S1.** Optical photo of 3D printed mold for circular SIN network incorporating rhombic intermediate cells between neighboring circular channels to preserve uniform channel width. The right panel shows a magnified view of the boxed area, highlighting the smooth continuity at the intersections.

**Figure S2.** Displacement contour plots (z-direction) obtained from finite element simulations for actuators with different microchannel geometries: (a) square, (b) circular, and (c) honeycomb patterns.

**Figure S3.** Schematic diagram about the wrinkle formation on the peaks of Ecoflex mold and its effect of bonding quality of PDMS layers

**Figure S4.** Photographs of the circular, square, and honeycomb patterned layers during tensile testing in the x and y directions. The x and y axes shown in each image represent the same conventionally assigned directions across all actuator designs, ensuring consistent and comparable deformation measurements under identical loading configurations.

**Figure S5.** Experimental setup used for bending measurements. The actuator is mounted in the testing frame, and the deflection and applied force are recorded using a laser displacement sensor and a load cell, respectively. These data are used to calculate the bending moment and curvature at the clamped end.

**Figure S6.** Schematic of the experimental setup for pressure-driven actuation and displacement measurement, showing the syringe pump, pressure sensor, laser displacement sensor, and DAQ system connected to the SIN actuator.

**Figure S7.** Boundary configurations used in the experiments: (a) free configuration, where the actuator is allowed to deform without external constraints, and (b) clamped configuration, where one edge of the actuator is fixed and deformation is measured at the opposite side.

**Figure S8.** Transient displacement response and corresponding pressure profile for the three SIN geometries (circular, square, and honeycomb) measured under clamped configuration during a pressurization–depressurization cycle: (a) x direction and (b) y direction.

**Figure S9.** Hysteresis loops (displacement–pressure) for (a) circular, (b) square, and (c) honeycomb actuators. The loop area reflects energy dissipation, while path separation indicates lag and flow resistance.

**Figure S10.** Schematic of the experimental setup for square-pulse actuation, showing the digital pump, solenoid valves, pressure sensors, Arduino controller, laser displacement sensor, and DAQ system connected to the SIN actuator.

**Figure S11.** Transient displacement and pressure response of the SIN actuators under a square-pulse pressure input, measured in free configuration for (a) circular and (b) honeycomb designs.

**Figure S12.** Blocking force as a function of applied pressure for the three SIN geometries (circular, square, and honeycomb), measured at the actuator’s central point for a 2 mm initial gap between the probe and the actuator.

**Figure S13.** Schematic illustration regarding effect of air chamber height on the bending moment.

**Figure S14.** Optical images of SIN actuators with different pattern resolutions (24×24, 18×18, 12×12, and 6×6 cells). In all designs, the ratio of channel width to bonding length and the total active area (2025 mm²) were kept constant, enabling isolation of the effect of cell density on actuation performance.

**Figure S15.** Magnified view of 12×12-square-cells actuators with different channel width. Channel widths systematically varied while keeping the sum of channel width and bonding length constant to isolate the influence of channel width on actuator performance.

**Figure S16.** Schematic diagram for measuring generated propulsion force by the movement of jellyfish actuator underwater.

**S.3 Energy Analysis of the Representative SIN Actuator**

For many classes of compliant and soft actuators, particularly those that store a substantial fraction of the input energy as elastic deformation, energy-based performance metrics are commonly defined using the elastic energy stored within the actuator.^[RS1, RS2]^

In general, the elastic strain energy stored in a deformable actuator can be expressed as:

$$U_{e}=\int_{V} \psi\text{ }dV$$

where $U_{e}$ is the total stored elastic strain energy, $V$ is the actuator volume, and $\psi$ is the strain energy density function. This formulation is generally applicable regardless of the constitutive model adopted for the material.

For thin deformable structures, the stored elastic energy may be further expressed in terms of the curvature field of the deformed surface using classical plate and shell theory:

$$U_{e}=\frac{1}{2}D\int_{A} \left( \kappa_{x}^{2}+2\nu\kappa_{x}\kappa_{y}+\kappa_{y}^{2}+2(1-\nu)\kappa_{xy}^{2} \right)dA$$

Where:

$$D=\frac{Eh^{3}}{12(1-\nu^{2})}$$

$D$ is the bending stiffness, $E$ is Young's modulus, $h$ is the actuator thickness, $\nu$ is Poisson's ratio, and $\kappa_{x}$, $\kappa_{y}$, and $\kappa_{xy}$ denote the principal and twisting curvatures of the deformed surface.

Although these equations establish the analytical framework for evaluating the stored elastic energy, their practical implementation requires the displacement data of entire actuator’s surface in order to determine the local curvature field at all points. Such information is not possible to acquire by experimental setup as the laser sensor only measures the displacement at a single point. Therefore, to avoid excessive simplifications and unrealistic analytical approximations, we extracted the stored elastic strain energy using a validated finite element analysis.

A finite element model was developed in COMSOL Multiphysics and validated against the experimentally observed deformations of the actuator exhibiting the largest displacement. The maximum deflection predicted by the simulation closely matched the experimentally measured displacement of the fabricated actuator. Moreover, the simulated deformed shape showed good agreement with that observed experimentally (Figure S17). These results indicate that the simulated strain energy provides a reliable estimate of the elastic strain energy stored in the physical actuator.

In addition, the pneumatic input energy was experimentally evaluated using:

$$U_{in}=\int P dV$$

where $P$ is the measured pressure and $V$ is the syringe-driven input volume.

Based on these values, we report two energy metrics. The first is the elastic power density, defined as:

$$\rho_{e}=\frac{U_{e}}{m t}$$

where $m$ is the actuator mass, and $t$ is the loading time required to reach the maximum applied pressure.

**Figure S17.** Deformation of the SIN actuator exhibiting the largest displacement. (a) Experimentally actuated, (b) Finite element prediction of the corresponding deformed configuration.

The second is elastic energy efficiency, defined as:

$$\eta_{e}=\frac{U_{e}}{U_{in}}$$

The first metric is closely related to the power density concept, while the second represents a measure of the actuator’s ability to convert pneumatic input energy into recoverable deformation energy.

The representative actuator achieved an elastic power density of 4.02 W/kg together with an elastic energy efficiency of 72.3% at a working pressure of 78 kPa. These results demonstrate that the SIN actuator exhibits favorable energy performance.

**S.4 Tables**

**Table S1.** Comparison of the SIN actuator and representative pneumatic soft actuators from the literature. Reference identifiers [35]–[42] are consistent with the citation numbering in the manuscript.

| Reference | Thickness [mm] | Bending Angle  [deg] | Normalized Bending Angle  [deg/mm] | Pressure  [kPa] |
| --- | --- | --- | --- | --- |
| SIN Actuator | 1.9 | 150 | 78.9 | 80 |
| Hao et al. [35] | 25 | 320 | 12.8 | 40 |
| Xiao et al. [36] | 3 | 200 | 66.7 | 35 |
| Gariya et al. [37] | 19 | 140 | 7.4 | 28 |
| Yu et al. [38] | 29 | 75 | 2.6 | 16 |
| Gonzalez et al. [39] | 10 | 120 | 12.0 | 172 |
| Gunawardane et al. [40] | 12 | 100 | 8.3 | 350 |
| Zhang et al. [41] | 15 | 160 | 10.7 | 130 |
| Liu et al. [42] | 26 | 109.3 | 4.2 | 60 |

**Table S2.** Comparison of the thrust performance of the proposed jellyfish-inspired SIN actuator with previously reported jellyfish-inspired robotic systems. Reference identifiers [44]–[47] are consistent with the citation numbering in the manuscript.

| Reference | Actuation mechanism | frequency [Hz] | Net thrust [mN] | Peak thrust [mN] | Bell diameter [mm] | Normalized peak thrust [mN/mm] | Normalized net thrust [mN/mm] |
| --- | --- | --- | --- | --- | --- | --- | --- |
| SIN Actuator | Pneumatic | 0.33 | 16.3 | 346 | 94 | 3.68 | 0.17 |
| Frame et al. [44] | Hydraulic | 0.40 | — | 560 | 210 | 2.67 | — |
| Matharu et al. [45] | TCP | 0.33 | 27 | — | 150 | 0.18 | 0.18 |
| Hu et al. [46] | Tendon-driven | 0.23 | — | 35.2 | 250 | 0.141 | — |
| Cheng et al. [47] | DEA | 1.60 | — | 0.12 | 156 | 0.0008 | — |

**References**

RS1. Tissaphern Mirfakhrai,  [John D.W. Madden](https://www.sciencedirect.com/author/7201575907/john-d-madden), and [Ray H. Baughman](https://www.sciencedirect.com/author/7202722728/ray-h-baughman), “Polymer artificial muscles,” *Materials Today* 10, no. 4 (2007): 30-38. <https://doi.org/10.1016/S1369-7021(07)70048-2>

RS2. Yoonho Kim, Hyunwoo Yuk, Ruike Zhao, Shawn A. Chester, and Xuanhe Zhao, “Printing ferromagnetic domains for untethered fast-transforming soft materials,” *Nature* 558, no. 7709 (2018): 274–279. <https://doi.org/10.1038/s41586-018-0185-0>
